# Efficacy and Safety of a Stabilized Composition of 26 mg/mL of High Molecular Weight Hyaluronic Acid for Aesthetic Applications

**DOI:** 10.3390/jcm14176015

**Published:** 2025-08-26

**Authors:** Basste Hadjab, Samuel Gavard Molliard, Jérémie Bon Bétemps, Marco Cerrano, Francesco de Boccard, Alexandre Finke

**Affiliations:** 1 Department of Research and Development, Kylane Laboratoires SA, 1228 Plan-les-Ouates, Switzerland; 2 Aesthetic Surgery Department, Clinique Entourage, 1003 Lausanne, Switzerland

**Keywords:** hyaluronic acid, sorbitol, injectable hydrogel, dermal filler, skin elasticity, aesthetic dermatology, clinical trial

## Abstract

**Background:** The growing demand for minimally invasive aesthetic procedures highlights the need for innovative injectable solutions that target skin aging beyond volumization. Hyaluronic acid (HA)-based fillers remain a cornerstone of aesthetic treatments, but traditional formulations often offer limited benefits in improving skin quality parameters such as elasticity. This study evaluated the efficacy and safety of an injectable formulation composed of 2.6% high molecular weight hyaluronic acid (H-HA) and 3.2% sorbitol, designed to improve skin biomechanical properties through subcutaneous administration. **Methods:** In this single-center, open-label, single-arm clinical trial, 86 participants aged 35 to 70 years received a single injection in the facial region. Clinical outcomes were assessed at baseline (day 0), one month (M1), and four months (M4) post-injection using the Global Aesthetic Improvement Scale (GAIS), instrumental Cutometer^®^ measurements of skin elasticity and recovery, and patient satisfaction questionnaires. **Results:** Significant improvements in skin elasticity and recovery were observed at M4 following a single administration. The product was well tolerated, with only mild and transient injection site reactions observed. **Conclusions:** These exploratory findings support the clinical benefit of combining high-concentration H-HA with sorbitol to enhance skin quality in a safe and minimally invasive manner. These preliminary results position this formulation as a promising option for facial rejuvenation, targeting biomechanical improvement through a single-session injectable protocol.

## 1. Introduction

Skin aging is a multifactorial process influenced by intrinsic factors, such as genetics and hormonal changes, as well as extrinsic factors, including ultraviolet radiation and pollution. These processes result in reduced collagen and elastin production, as well as diminished levels of hyaluronic acid (HA), a key component of the extracellular matrix (ECM). The progressive decline in HA levels leads to dehydration, loss of elasticity, and the appearance of wrinkles and sagging [1,2,3]. Hyaluronic acid, a naturally occurring glycosaminoglycan, plays a vital role in maintaining skin hydration, elasticity, and structural integrity. In the context of aesthetic medicine, HA-based treatments have become a cornerstone for addressing signs of aging, offering solutions for both volumization and skin quality improvement [4,5,6]. Traditional HA fillers typically rely on crosslinking agents, such as 1,4-butanediol diglycidyl ether (BDDE), to enhance product durability and stability. While BDDE-crosslinked HA products for skin quality improvement have proven effective for durable treatments [7], concerns regarding stiffness, unexpected late swelling, and inflammatory reactions [8,9] have prompted the development of alternative stabilization techniques. On the other hand, HA-based skin quality enhancers are typically low crosslinked or non-crosslinked, resulting in reduced volumizing effects and shorter durations of action. These products, injected in the dermis, primarily focus on skin rejuvenation by enhancing protein expression at the dermal-epidermal junction, effectively targeting fine lines and improving skin hydration [10]. In this context, a novel stabilized 2.6% high molecular weight hyaluronic acid (H-HA)/3.2% sorbitol formulation represents an innovation in HA-based injectables. Specifically designed for subcutaneous injection, this composition combines 26 mg/mL of H-HA stabilized with 32 mg/mL of sorbitol, a highly biocompatible molecule, in a phosphate buffer solution. Unlike crosslinking methods, the stabilization of this formulation relies on the formation of extensive low-energy hydrogen bonds between sorbitol and HA. This approach results in a flexible gel that mimics the behavior of surrounding tissues, creating a dynamic HA gel with a unique combination of elasticity and cohesivity [11].

Compared to existing non-crosslinked or low-crosslinked HA products designed for dermal injection, this new formulation offers a distinct profile in terms of injection depth, concentration, and potential duration of effect. While most skin quality enhancers are injected intradermally [12,13], this product is administered subcutaneously. The subcutaneous route offers improved patient comfort and making it particularly suitable for broader coverage and deeper tissue integration.

Furthermore, studies have demonstrated the appropriate distribution and high biocompatibility of this stabilized 2.6% H-HA/3.2% sorbitol composition in adipose tissue. It has shown efficacy in improving skin biomechanical properties, including firmness and elasticity, while remaining well-tolerated [14]. Additionally, this formulation has been shown to enhance biological markers of skin quality in the dermis following injection into the superficial adipose tissue. Evidence supports its ability to stimulate the endogenous production of key skin components, including HA, elastin, collagens, and fibrillin [15]. Its composition may contribute to a prolonged residence time by resisting enzymatic degradation without introducing chemical crosslinkers [11].

Together, these properties also expand the indications to include areas with mild to moderate laxity, such as the arms, which are not commonly addressed by standard dermal injectables. From a clinical perspective, this broader versatility and durability, combined with the absence of BDDE and a lower risk of delayed inflammatory reactions, positions the product as a promising alternative for patients seeking subtle, natural improvements in both facial and body skin quality [10].

The aim of this study was to evaluate the clinical efficacy and safety of 2.6% H-HA/3.2% sorbitol injectable devices in aesthetic applications, focusing on skin quality improvement for both facial and body treatments. The primary objective was to assess aesthetic outcomes using the Global Aesthetic Improvement Scale (GAIS). Secondary objectives included subjective assessments evaluating injector and patient satisfaction, as well as objective improvements in skin elasticity through objective metrics. Additionally, the safety profile of the 2.6% H-HA/3.2% sorbitol products was documented.

## 2. Materials and Methods

### 2.1. Study Design

This study was a monocenter, open-label clinical trial designed to evaluate the efficacy, safety, and patient satisfaction of 2.6% H-HA/3.2% sorbitol injectable hyaluronic acid devices (Kylane Laboratoires, Geneva, Switzerland) for facial and body rejuvenation. The study was conducted in compliance with internationally recognized standards, including ISO 14155:2020 guidelines for clinical investigation of medical devices [16] and the principles outlined in the Declaration of Helsinki. The open-label design was chosen to allow for comprehensive data collection in a real-world clinical setting, enabling investigators to observe treatment outcomes across various anatomical sites while adhering to standardized protocols. The study adhered to internationally recognized clinical research standards and received necessary approvals prior to its initiation. The study protocol and associated documents were reviewed and approved by an independent Ethics Committee to ensure that the rights, safety, and well-being of participants were protected throughout the trial. Approval was obtained from the Agence Nationale de Sécurité du Médicament et des Produits de Santé (ANSM), the French National Health Authority, which oversees the regulation of medical devices and clinical investigations in France. This approval confirmed that the study complied with national and European Union requirements for clinical investigations involving medical devices. The study took place at a specialized aesthetic dermatology center in France over six months, from the initial intervention to the final follow-up. Data were collected at three key time points: baseline (day 0), month 1 (M1), and month 4 (M4), with an additional safety assessment at month 6 (M6). This timeline was designed to evaluate both the immediate and sustained effects of the treatment, highlighting its potential for long-lasting efficacy.

### 2.2. Participants

The study recruited 86 participants aged 35–70 years who sought aesthetic enhancements for facial and body rejuvenation. Participants were recruited through advertisements in local aesthetic clinics and via word-of-mouth referrals. During the screening phase, potential participants underwent a thorough medical history review and physical examination to ensure eligibility. Baseline assessments included:

- Standardized photographs of treatment areas.
- Initial measurements of skin elasticity (Cutometer^®^).
- Completion of a demographic and lifestyle questionnaire to control for confounding factors such as smoking and UV exposure.

Participants were carefully selected to ensure a homogeneous study cohort while reflecting a diverse population. Selection was conducted based on predefined inclusion and exclusion criteria to minimize potential confounding factors. Eligible participants were healthy males and females aged 35–70 years, seeking visible improvement of facial (malar or submalar) or body skin quality (neck or décolleté), depending on group assignment. For facial treatment, participants were required to present clinical signs of skin dryness, as evaluated during the screening visit. Exclusion criteria included a history of procedures involving active dermal responses in the treated areas within the past six months or prior injections of permanent or semi-permanent fillers in the targeted zones in the last 18 months. Additionally, participants were excluded if they had undergone intensive UV exposure. Participants were divided into two groups based on the target treatment areas, with specific mandatory and optional regions for each group.

In group 1, 64 participants were injected in facial areas: malar (cheek), submalar (below the cheekbone), and optionally in mandibular (jawline) and chin areas. The objective was to improve skin quality, hydration, and elasticity in the midface and optional lower facial areas, addressing fine lines, skin laxity, and volume loss. The malar and sub-malar regions are central to midface aesthetics and play a critical role in restoring facial harmony. Optional areas, such as the chin and jawline, complement the midface improvements, offering a balanced and rejuvenated appearance.

In group 2, 22 participants were injected in different body areas: décolletage (upper chest area), neck, and optionally in the abdomen and internal sides of the arms. The objective was to improve skin quality, hydration, and elasticity in body regions prone to laxity and dehydration, addressing age-related changes. The décolletage and neck are highly visible areas prone to dehydration and laxity, making them ideal targets for body rejuvenation. Optional areas, including the abdomen and inner arms, address patient-specific concerns in regions that benefit from enhanced elasticity and hydration.

### 2.3. Intervention

The intervention in this study involved a single treatment of 2.6% H-HA/3.2% sorbitol injectable devices, specifically formulated for aesthetic applications targeting facial and body skin rejuvenation and designed to be injected into the subcutaneous tissues. The subcutaneous injection techniques were tailored to the anatomical and functional requirements of each treatment area at the discretion of the investigator using the following techniques: linear threading, retrograde injections, cross-hatching, and fanning techniques. 27G and 30G needles were used as well as a 25G canula. A mean quantity of 1.1 ± 0.5 mL per treated area was injected in the face (malar/submalar, mandibular, chin), while 3.7 ± 1.6 mL was injected in treated body areas (neck/decolletage, arms, abdomen). Skin was cleansed using (e.g., chlorhexidine or alcohol-based antiseptic) prior to injection. Treated areas were gently massaged only if needed to ensure an even distribution. Subjects were advised to avoid strenuous activity, direct sun exposure, and application of cosmetics on the treated area for 24 h post-injection. All injection procedures were performed by experienced, board-certified aesthetic practitioners trained and standardized on the study protocol prior to subject enrollment.

### 2.4. Endpoints

The endpoints of this study were meticulously designed to provide a robust assessment of the clinical performance, safety, and patient satisfaction associated with 2.6% H-HA/3.2% sorbitol injectable hyaluronic acid (HA) devices. These endpoints included a mix of subjective assessments, objective measurements, and safety evaluations, ensuring a holistic evaluation of the intervention’s efficacy and tolerability.

The primary objective was to assess aesthetic improvement using the GAIS as rated by the investigator. Secondary objectives included evaluating the impact of 2.6% H-HA/3.2% sorbitol on skin biomechanical properties, assessing participant satisfaction through validated questionnaires, and monitoring safety through adverse event reporting.

#### 2.4.1. Global Aesthetics Improvement

The aesthetic improvement was assessed at each follow-up visit for each treated area concerned using the GAIS. This 5-level scale ranging from 1 (very much improved) to 5 (worse), widely utilized in aesthetic studies, evaluates the degree of improvement in appearance post-treatment, as perceived by investigators (iGAIS) or subjects (sGAIS). The GAIS responder rate was defined as the proportion of participants achieving a GAIS score of 1 (very much improved), 2 (much improved), or 3 (improved). This threshold reflects clinically meaningful improvements in aesthetic outcomes.

#### 2.4.2. Satisfaction

Participant satisfaction was evaluated using a validated questionnaire to assess perceived treatment outcomes, comfort, and overall experience. This questionnaire was administered at one month (M1) and four months (M4) post-treatment to capture subjective perceptions of benefits and satisfaction. Participants rated the aesthetic outcomes of treated areas, such as hydration, smoothness, or elasticity of the skin, using a 5-point Likert scale ranging from “very dissatisfied” (1) to “very satisfied” (5). Injector satisfaction with the quality of the injection procedure was assessed immediately after treatment (D0) using a subjective evaluation questionnaire. This allowed for feedback on ease of use and injection handling.

#### 2.4.3. Skin Biomechanical Properties

**Figure 1:**
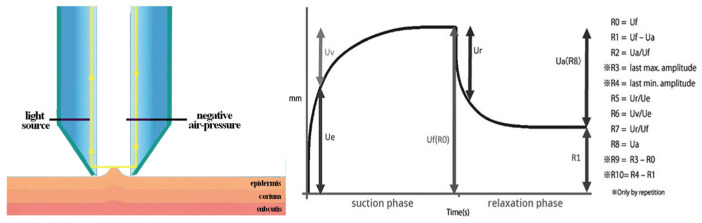
Scheme and typical curve from a Cutometer^®^ measurement.

Skin elasticity was evaluated on the face using the Cutometer^®^-MPA 580 Cutometer^®^ (Courage & Khazaka, Köln, Germany), a non-invasive device that measures the biomechanical properties of the skin through suction-based deformation. The penetration depth is measured using a non-contact optical system. This system captures the skin’s deformation and recovery behavior. The ability of the skin to return to its original position is represented as a curve, which is then analyzed using specialized software to calculate key biomechanical parameters (Figure 1). Assessment of skin elasticity was obtained through the skin biological elasticity (R7) which represent the proportion (in %) of the immediate recovery (Ur) compared to the total amplitude after suction (Uf). Another key parameter, residual deformation (R1), measures the skin’s ability to return to its initial state at the end of the recovery phase (Uf – Ua, in mm). The opposite parameter -R1 represents global recovery on an increasing scale. The secondary endpoints were defined as changes from baseline (day 0) of these viscoelastic parameters at M1 and M4 to capture short-term and long-term changes. Three measurements were performed per patient and per standardized treatment site (malar region) to ensure consistency.

#### 2.4.4. Safety

Safety outcomes were a critical focus of the study, aimed at evaluating the tolerability and safety profile of 2.6% H-HA/3.2% sorbitol injectable hyaluronic acid (HA) devices for both facial and body treatments. The assessment of adverse events (AEs) and injection site reactions (ISRs) emphasized their frequency, severity, duration, and potential relationship to the investigational product. At each time point, the investigator (or injector on day 0) evaluated local tolerance using a predefined, standardized 4-point scale for each expected sign (i.e., redness, pain/tenderness, induration, swelling, lumps, bruising, itching, and discoloration). This scale is aligned with recognized clinical standards for ISR assessment: Grade 0 (none)—no symptoms observed; Grade 1 (mild)—discomfort noted, but without disruption to normal daily activities and no treatment considered; Grade 2 (moderate)—discomfort sufficient to reduce or affect normal daily activities, with treatment possibly needed; and Grade 3 (severe)—inability to work or to carry out normal daily activities, with treatment required. The safety of the 2.6% H-HA/3.2% sorbitol range was assessed through:

- Daily logs: Participants recorded immediate and early ISRs daily until M1.
- Injector reports: Immediate ISRs were documented by the injector immediately after treatment.
- Independent evaluations: ISRs were evaluated by an independent investigator at M1 and M4.
- Adverse event monitoring: AEs were collected and recorded throughout the study until M6.

### 2.5. Statistical Analysis

#### 2.5.1. Sample Size

A minimum sample size of 78 participants was required to detect a significative difference of at least 15% from the pre-defined 60% threshold in GAIS responder rates at M1 with 80% power at a 5% significance level. Considering a potential drop-off of 10%, a minimum of 86 subjects were included in the study.

#### 2.5.2. Statistical Method

All performance and safety analyses were based on the full analysis set of 86 subjects who completed the study. Statistical tests were performed using SAS 9.4 (POWER procedure) to assess the change from baseline, if applicable. For qualitative variables based on self-assessment questionnaires, such as aesthetic improvement and satisfaction for both investigator and subjects, the proportion of satisfied participants (%) and 95% confidence interval (95%CI) calculated using the Clopper Pearson method are displayed. For biomechanical parameters, a two-tailed paired *t*-test with a significance level α of 0.05 is carried out to assess whether each time point after injection (M1, M4) differed significantly from the baseline value (D0). To check the Gaussian distribution assumption, a Shapiro–Wilk test (α = 0.01) was performed. In case of rejection, a two-tailed Wilcoxon signed rank test was used. In both cases, significance was visually rated on graphics. A Bonferroni correction was applied to account for multiple comparisons, considering two timepoints (M1 and M4) versus baseline for each Cutometer parameter. Consequently, the significance threshold α was adjusted to 0.025 per parameter (ns: *p*-value > 0.025; *: *p*-value ≤ 0.025; **: *p*-value ≤ 0.005; ***: *p*-value ≤ 0.0005). Summaries (number, N; mean; standard deviation, SD; 95% confidence interval, 95%CI) were calculated for the values and changes from baseline.

## 3. Results

The study enrolled a total of 86 participants, all of whom met the inclusion criteria and underwent baseline assessments. No withdrawals due to adverse events occurred and all of them completed the study.

Participant characteristics are summarized in Table 1. Subjects included in group 2 were older on average (57.1 ± 9.9 years) than subjects included in group 1 (52.7 ± 9.7 years).

### 3.1. Global Aesthetic Improvement Scale

**Figure 2:**
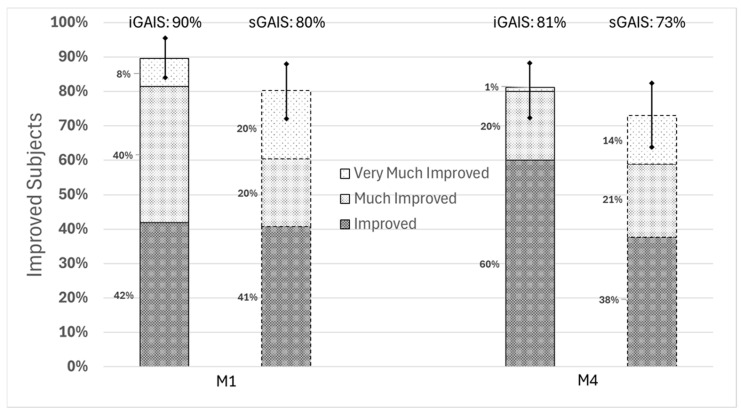
iGAIS and sGAIS one month and four months after the single session of injection for all participants.

From a global point of view, the primary endpoint results showed that after a single injection of 2.6% H-HA/3.2% sorbitol range, the proportion of responder (subjects with a GAIS “Very Much Improved”, “Much Improved”, or “Improved”) for the GAIS evaluated by the independent investigator (iGAIS) at M1 was 90% [95%CI (81%, 95%]). This percentage was calculated by summing the total number of subjects from both groups falling into these three categories and dividing by the total number of subjects included in the analysis. At M4, a persistent proportion of subjects was still responding with a iGAIS of 81% [95%CI (71%, 89%)], as can be seen in Figure 2 and Figure S1 and Tables S1 and S2. The GAIS self-reported by subjects (sGAIS) consistently improved from baseline at M1 and M4 with 80% [95%CI (70%, 88%)] and 73% [95%CI (62%, 82%)], respectively.

The aesthetic improvements of treated areas for the whole 2.6% H-HA/3.2% sorbitol range as assessed by both investigator and subjects were remarkably high. One month after injection (M1), 85% [95%CI (77%, 92%)] of treated areas were considered improved according to iGAIS, while 80% [95%CI (70%, 87%)] were rated at least improved by subjects (sGAIS). At M4, these proportions slightly decreased to 78% [95%CI (68%, 86%)] and 70% [95%CI (60%, 79%)], respectively.

**Figure 3:**
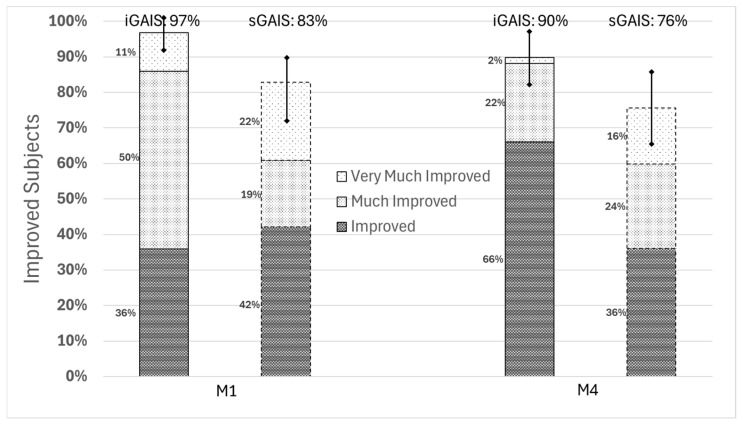
iGAIS and sGAIS one month and four months after the single session of injection in the face.

Focusing on the injections on the face area (Figure 3), the results at M1 are particularly notable with 97% [95%CI (89%, 100%)] of subjects rated at least “improved” at M1 by the investigator and 90% [95%CI (80%, 96%)] at M4. GAIS ratings by investigators were slightly higher than sGAIS ratings.

### 3.2. Patient and Injector Satisfaction Outcomes 

Participants consistently noted high global satisfaction scores (77% [95%CI (67%, 84%)] at M1, 67% [95%CI (56%, 77%)] at M4) closely aligned with investigator-assessed GAIS ratings, underscoring the alignment of clinical and patient-perceived outcomes.

Even four months after a single injection, more than two-thirds of the subjects reported sustained improvements in hydration (69% [95%CI (58%, 78%)] at M1, 71% [95%CI (60%, 80%)] at M4), firmness (64% [95%CI (53%, 74%)] at M1, 67% [95%CI (56%, 77%)] at M4), and elasticity (73% [95%CI (63%, 82%)] at M1, 67% [95%CI (56%, 77%)] at M4). Additionally, a smoother skin texture was frequently noted by approximately 69% of subjects at both M1 (67% [95%CI (56%, 77%)]) and M4 (69% [95%CI (58%, 78%)]). The reduction of fine lines after four months was the only parameter below the 60% threshold (56% [95%CI (45%, 67%)]), although this filling behavior was more widespread at M1 (64% [95%CI (53%, 74%)]).

For all indications of 2.6% H-HA/3.2% sorbitol, the injectors were highly satisfied with handling comfort and easiness of product positioning for 100% [95%CI (95%, 100%)] of the subjects. The level of satisfaction quoted by injectors was barely lower either immediately or after massage on 99% [95%CI (94%, 100%)] and 98% [95%CI (92%, 100%)] of the subjects, respectively.

### 3.3. Facial Skin Biomechanical Properties

**Figure 4:**
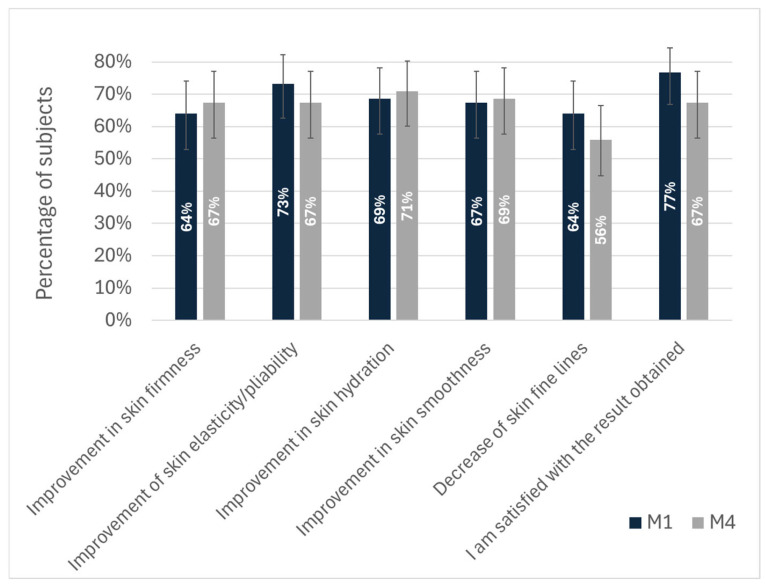
Efficacy evaluation by participants performed at M1 and M4.

Data from 64 subjects injected in the face (group 1) were analyzed (Figure 4 and Table S3). Both mean skin biological elasticity (R7) and global recovery (-R1) decreased one month after treatment but not significantly (*p*-value > 0.0125).

**Figure 5:**
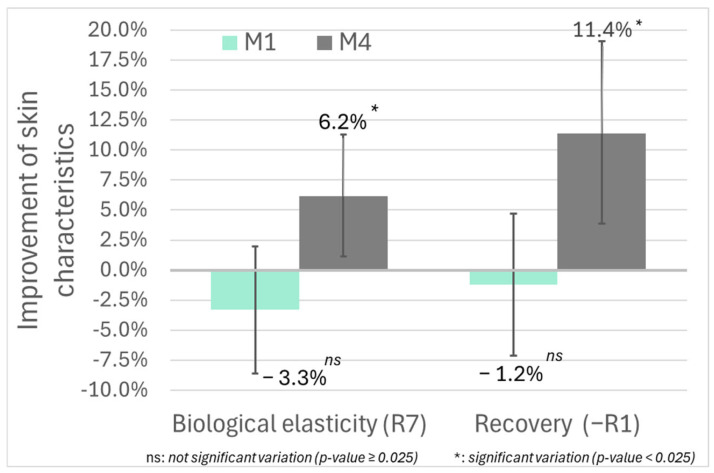
Time-dependent variations in skin biomechanical properties measured by Cutometer.

At M4, improvement from baseline was significant for both parameters. With a mean R7 value of 0.372 [SD = 0.086; 95%CI (0.350, 0.394)] at baseline to 0.395 [SD = 0.079; 95%CI (0.375, 0.415); *p*-value = 0.0132], biological elasticity was significantly increased to 6.2. In the same manner, recovery increased by 11.4%; likewise, the mean residual deformation after suction decreased from 0.140 [SD = 0.050; 95%CI (0.127, 0.152)] at baseline to 0.124 [SD = 0.037; 95%CI (0.114, 0.133); *p*-value = 0.0097] at M4 (Figure 5).

In the absence of a validated minimal clinically important difference (MCID) for Cutometer parameters, an exploratory anchor-based analysis was conducted using patient-reported satisfaction as a reference. Mean improvements in R7 (elasticity) and -R1 (recovery) were compared across patient satisfaction groups, with respective self-reported global satisfaction in elasticity and firmness serving as the external anchor. While this approach does not establish clinical validity, it offers preliminary insights into potential thresholds of perceptible change. The difference in Cutometer values between the “no change” group and the “satisfied” or “very satisfied” groups was used as a proxy for the MCID. For R7, the mean differences were 3.4% (“satisfied”) and 3.8% (“very satisfied”). For -R1, the corresponding differences were approximately 6.9% and up to 10.5%, respectively. Using the lowest of these estimates as the MCID threshold, 44% and 48% of participants showed substantial improvements in skin elasticity and firmness, respectively. These estimates may help guide future studies aiming to determine clinically meaningful changes in skin biomechanics.

### 3.4. Safety Assessment

For the 2.6% H-HA/3.2% sorbitol devices for both facial and body treatments, injection site reactions (ISRs) were reported by the investigator immediately after injection in all but one subject. The ISRs observed—such as redness, bruising, and swelling—were fully consistent with those commonly associated with HA-based injectable gels (Figure S2). Most reactions were mild (85.8%) or moderate (14.1%). During the first month post-injection, 59% of subjects reported at least one ISR following facial injections, while 90% reported ISRs after body injections. Most reactions resolved within seven days, and no signs were observed by subjects or reported by the investigator one month and four months post-injection. A total of 68 adverse events (AEs) were reported in 32 subjects during the 6 months duration of the study, representing 37% of the study population. Among these, only four were possibly related to the studied device (adverse device effects, ADEs), which included two cases of pain, one migraine, and one headache all reported less than 4 h after injection. All ADEs resolved without sequelae by the study’s conclusion.

## 4. Discussion

The results of this study demonstrate that a single injection of 2.6% H-HA/3.2% sorbitol injectable devices, utilizing a sorbitol-stabilized HA formulation, provides significant and sustained improvements in skin elasticity and overall aesthetic appearance. The primary objective of demonstrating aesthetic improvement using the GAIS was achieved, with both investigator and participant ratings confirming significant improvements across facial and body treatment areas. Responder rates scored by the investigator of 90% at M1 for all treatments combined underscore the versatility and adaptability of 2.6% H-HA/3.2% sorbitol to diverse anatomical areas. Notably, the improvements assessed by iGAIS on group 1 (facial treatment) surpass those reported for many comparable products on the market when injected in a single session, with up to 97% satisfaction [17,18,19,20]. The age distribution might explain subtle differences in responder rates with group 2 (body treatment), whose participants were older on average than group 1. Generally, aesthetic expectations and needs increase with age [21,22], with necessary tailored strategies.

Despite the absence of crosslinking in 2.6% H-HA/3.2% sorbitol and the lack of touch-up treatments that are traditionally proposed to enhance durability and effectiveness, aesthetic improvements were remarkably sustained at M4, with 81% of responders in general according to iGAIS, and still about 90% for subjects of the group 1. The sorbitol-stabilized formulation likely contributed to this longevity, as previously evidenced in in vitro studies [11]. These findings highlight the efficacy of this formulation in achieving long-lasting results with minimal intervention. A slight reduction in improvement scores between M1 and M4 was noted, reflecting the gradual dissipation of the treatment effect, as expected for resorbable HA formulations.

These reported subjective outcomes are supported by robust objective measurements of skin quality. Loss of elasticity is a hallmark of aging skin, with both aesthetic and functional implications. Assessment of skin elasticity provides valuable insights into the rejuvenation effects of treatment. The Cutometer^®^, widely utilized in aesthetic studies, was employed to evaluate key parameters, including global recovery (-R1) and biological elasticity (R7), which reflect the skin’s capacity for resilience and flexibility. A higher value of -R1 (closer to 0), measured as the final deformation after suction, signifies better global recovery, whether through immediate elasticity or delayed viscoelasticity. This metric is particularly relevant in assessing skin laxity, as a lower ability to recover its shape is associated with sagging and structural weakening over time. Biological elasticity (R7) is calculated as the ratio of immediate recovery (Ur) to maximum deformation (Uf), with higher values (closer to 1) indicating better elasticity and dynamic recovery. These parameters correlate strongly with age and overall skin health, as reported in multiple studies [21,23,24,25,26].

In this study, a non-significant initial decrease in biomechanical parameters was observed at M1. A similar behavior has already been observed with other injectables [26]. A plausible explanation for this short-term effect is the hydrophilic nature of the injected gel. The high intrinsic water content and strong water-binding capacity of the 2.6% H-HA/3.2% sorbitol formulation may transiently dilute or disorganize the local elastic fiber network of the skin in the treated areas. The late clinical improvements at M4 were consistent with patient-reported outcomes and aligned with the expected biological activity of the product. Significant increases in skin recovery (up to +11.4%) and elasticity (up to +6.2%) were recorded using the Cutometer^®^, underlining the rejuvenating potential of the formulation. Notably, the proportion of participants showing clinically meaningful improvements in biomechanical skin properties (44–48%) was comparable to that reported for hydration by HA-based skin booster products, even when including optional touch-up treatments [20]. However, it should be noted that the thresholds for clinically meaningful improvements were estimated from an exploratory anchor-based analysis and require further validation in future studies.

These results are biologically plausible and consistent with the known mechanisms of action of HA-based injectables. The gradual improvement in elasticity observed from baseline to M4 mirrors the delayed onset of clinical benefits documented for similar non-crosslinked HA formulations [27,28]. This delay has been attributed to the bio-stimulatory properties of HA, which promote fibroblast activation, extracellular matrix synthesis, and dermal tissue remodeling [29,30]. Specifically, HA is known to stimulate the endogenous production of structural proteins such as elastin, fibrillin, and multiple types of collagen.

Although molecular upregulation of these elements has been demonstrated shortly after injection—particularly in a previous study for the 2.6% H-HA/3.2% sorbitol formulation [15]—the formation of mature and functional extracellular matrix structures takes time. For example, newly synthesized collagen is initially deposited in a random, disorganized fashion during granulation. It is then progressively remodeled and aligned to restore dermal tensile strength and viscoelasticity—a process that can span several months [31]. The elasticity improvements measured at M4 likely reflect the culmination of these complex regenerative processes. These results are consistent with the literature, where linear hyaluronic acids supplemented with PRP or niacinamide have been shown to progressively improve biomechanical parameters, particularly R7, over time following one or more injections [32,33].

Due to the nature of non-crosslinked or lightly stabilized hyaluronic acid formulations, touch-up and repeat treatments are generally expected to maintain and enhance clinical outcomes over time [12,20,27]. Although the sorbitol-based stabilization improves resistance to enzymatic degradation relative to standard non-crosslinked HA, the effect is not permanent, necessitating periodic maintenance injections to sustain improvements in skin quality. The subcutaneous mode of administration and enhanced durability may allow for longer intervals between sessions compared to typical intradermal injections of low-crosslinked HA, potentially improving patient adherence and satisfaction. Further longitudinal studies are needed to establish optimal retreatment schedules and to evaluate cumulative benefits and safety in long-term use. This compares favorably with other linear supplemented HA, which requires three sessions spaced three weeks apart to achieve and maintain results [34]. The other clinical evaluation enrolled subjects aged 35–55 years. In our trial, we selected a broader and older age range (35–70 years), a group typically characterized by diminished regenerative capacity and more advanced photoaging factors, which may reduce responsiveness to treatment [35].

The tolerability of 2.6% H-HA/3.2% sorbitol was excellent, with no serious adverse events (SAEs) reported. Most adverse events (AEs) were mild and transient, consistent with the absence of BDDE crosslinker and/or the addition of other chemical manufacturing aid. Only 59% of participants reported ISRs between D0 and M1. No ISRs were reported from M1 to M6 by any subject.

High satisfaction rates, ranging from 60–75% at M1 and M4, reflect the combined performance and safety of the product and validate the treatment’s acceptability and perceived efficacy from the participant’s perspective. Participants highlighted improvements in hydration, firmness, and smoothness, with satisfaction levels remaining stable between M1 and M4. These outcomes are consistent with those reported for skin boosters and skin quality enhancers after a single injection [14,17], further validating the efficacy of 2.6% H-HA/3.2% sorbitol.

Despite its promising results, this study has several limitations. The absence of a direct comparator group limits the ability to contextualize the performance of 2.6% H-HA/3.2% sorbitol relative to other HA-based treatments. Although the four-month follow-up demonstrated sustained improvements, longer-term data are needed to evaluate durability and optimal retreatment intervals. To confirm and strengthen these preliminary findings, future studies should include randomized controlled trials with active comparators. Such trials would provide a higher level of evidence by minimizing bias and allowing for definitive assessment of the product’s efficacy against existing standards of care.

Another limitation lies in the relatively limited characterization of baseline skin quality, which could affect the interpretation of observed changes. Incorporating more detailed assessments (e.g., histological data, imaging, or validated grading scales) at baseline would improve the precision of future evaluations.

In addition, the use of multiple endpoints across several timepoints introduces a risk of type I error inflation. While exploratory in nature, the results should be interpreted with caution, and future studies may consider appropriate statistical corrections or adjusted thresholds for significance.

Finally, four authors are employees of the sponsoring company that manufactures the study product. Although this presents a potential conflict of interest, the study design incorporated objective measurements (Cutometer^®^ parameters), standardized procedures, and blinded patient-reported outcomes to help mitigate bias and ensure the integrity of data interpretation.

These first clinical exploratory results on 2.6% H-HA/3.2% sorbitol indicate that this stabilized HA gel represents a significant advancement in HA-based aesthetic treatments. The sorbitol-stabilized formulation ensures smooth tissue integration. This makes 2.6% H-HA/3.2% sorbitol particularly suitable for patients seeking natural and subtle aesthetic enhancements. Its versatility extends beyond facial applications to body areas such as the neck, décolletage, and optional zones (e.g., inner arms, abdomen), addressing a growing demand for comprehensive aesthetic solutions. With extended duration and minimal risk, 2.6% H-HA/3.2% sorbitol offers a compelling option for patients seeking non-surgical rejuvenation.

## 5. Conclusions

This exploratory study highlights 2.6% H-HA/3.2% sorbitol as a safe, effective, and versatile solution for aesthetic skin rejuvenation. A single injection of this sorbitol-stabilized, BDDE-free HA formulation delivers significant improvements in elasticity, and overall appearance, sustained for up to four months, with high participant satisfaction (73.7% at M1) and 100% injector satisfaction regarding product handling. These promising results position 2.6% H-HA/3.2% sorbitol as a valuable addition to the armamentarium of minimally invasive aesthetic treatments, offering natural and subtle enhancements across both facial and body applications.

Future studies with larger, more diverse populations, comparative arms, and longer follow-up periods will further validate these exploratory findings, optimize treatment intervals, and expand 2.6% H-HA/3.2% sorbitol’s clinical applications.

## Figures and Tables

**Table 1:** General characteristics of the participants.

Parameter	Total	Group 1: Face	Group 2: Body
Number N	86	64	22
Age (mean ± SD)	53.8 ± 9.9	52.7 ± 9.7	57.1 ± 9.9
**Gender Distribution (%)**			
Female	88.4%	85.9%	95.5%
Male	11.6%	14.1%	4.5%

## Data Availability

The data presented in this study are openly available within the article files.

## Supplementary Materials

The following supporting information can be downloaded at: https://www.mdpi.com/article/10.3390/jcm14176015/s1, Figure S1: Images from three selected study patients (i.e., from left [D0], pre-baseline to right [M1 and M4]). (A1–A3) Patient N° G1-01. (B1–B3) Patient N° G1-02. (C1–C3) Patient N° G1-04; Figure S2: Distribution of injection site reaction (ISR) severity at day 0. ISR types include bruising, discoloration, induration/firmness, itching, lumps/bumps, pain/tenderness, redness, and swelling, categorized according to severity grades (mild, moderate, severe); Table S1: Investigator GAIS results; Table S2: Subject GAIS results; Table S3: Skin texture parameters parameter (i.e., Uf and Ur) values for each subject.

## Abbreviations

The following abbreviations are used in this manuscript:

ADEs	Adverse Device Effects
AEs	Adverse Events
ANSM	Agence Nationale de Sécurité du Médicament et des produits de santé
BDDE	1,4-Butanediol Diglycidyl Ether
GAIS	Global Aesthetic Improvement Scale
HA	Hyaluronic Acid
iGAIS	Investigator Global Aesthetic Improvement Scale
ISR	Injection Site Reaction
sGAIS	Subject Global Aesthetic Improvement Scale
UV	Ultraviolet
