# Supplementary material for: Efficacy and Safety of a Stabilized Composition of 26 mg/mL of High Molecular Weight Hyaluronic Acid for Aesthetic Applications
Source: J Clin Med. 2025 Aug 26;14(17):6015. doi: 10.3390/jcm14176015 (PMC12428844; doi:10.3390/jcm14176015)
Supplement: Supplementary file 1 [file jcm-14-06015-s001.zip › jcm-3785815-supplementary.pdf]

Journal of Clinical Medicine

MDPI

Supplementary Materials:

Efficacy and Safety of a Stabilized Composition of 26 mg/mL of High Molecular Weight Hyaluronic Acid for Aesthetics Applications

Basste Hadjab*, Samuel Gavard Molliard, Jérémie Bon Betemps, Marco Cerrano, Francesco de Boccard, Alexandre Finke

1. Supplementary Figures

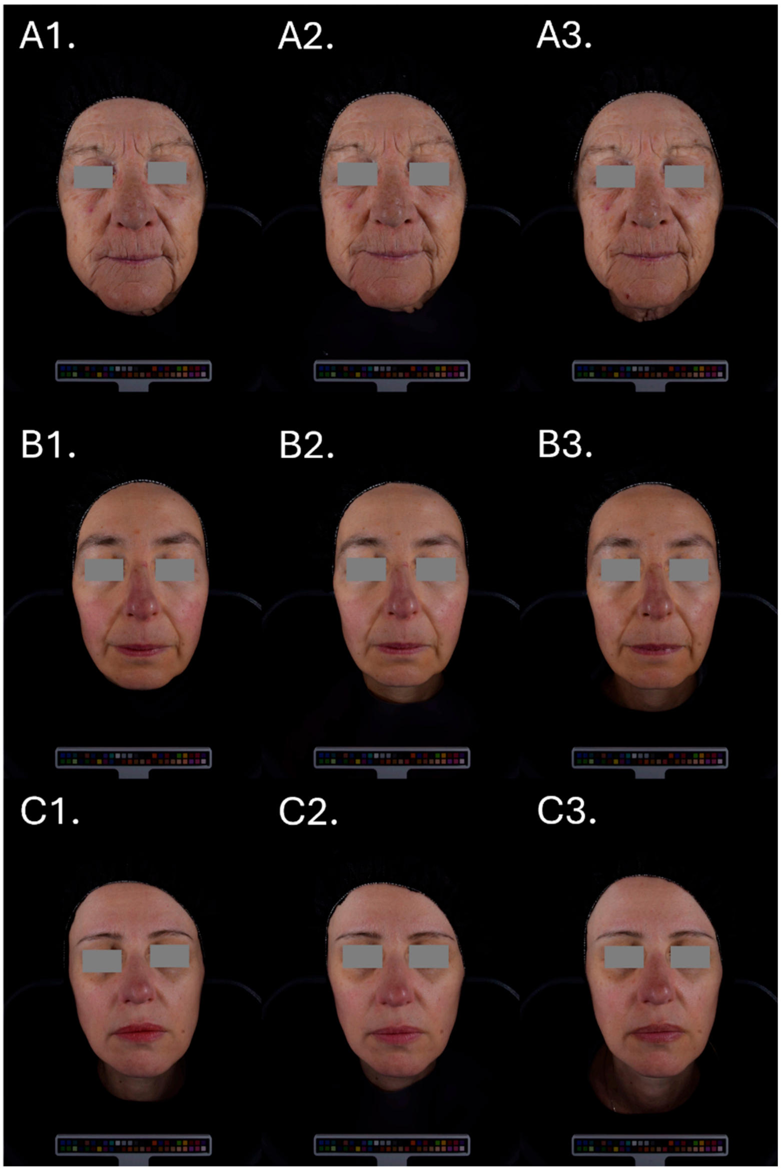

Figure S1. Images from three selected study patients (i.e., from left [D0], pre-baseline to right [M1 and M4]). (A1-A3) Patient N° G1-01. (B1-B3) Patient N° G1-02. (C1-C3) Patient N° G1-04.

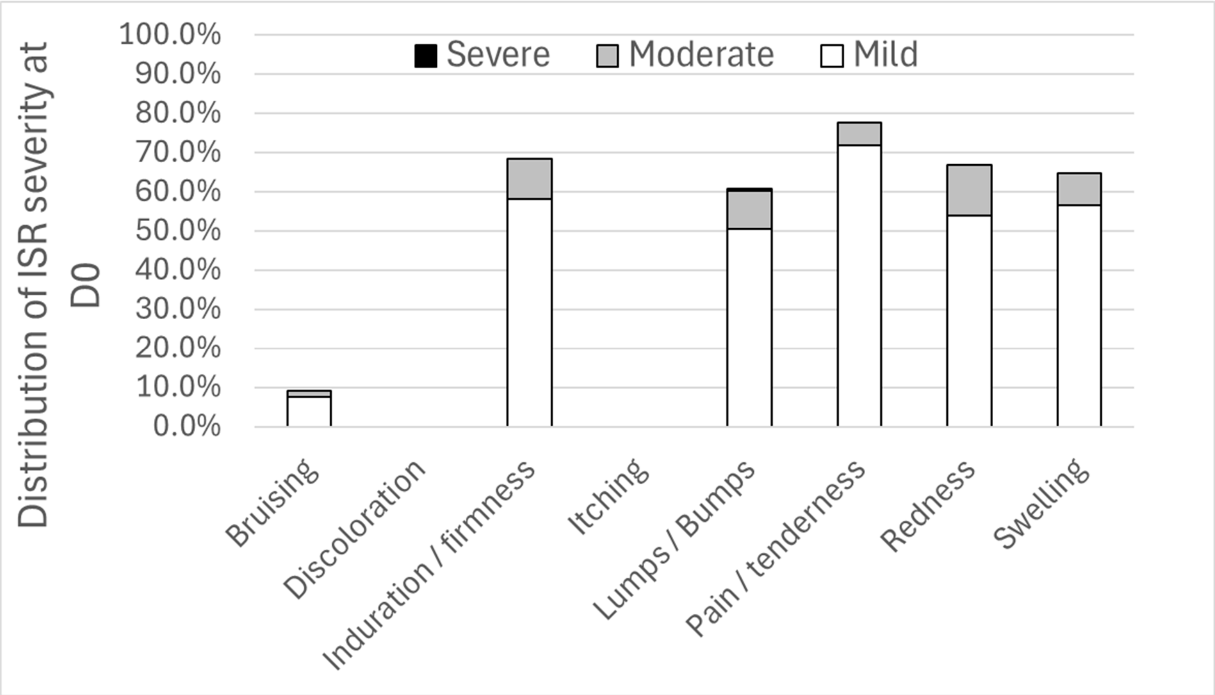

FigureS2: Distribution of injection site reaction (ISR) severity at Day 0. ISR types include bruising, discoloration, induration/firmness, itching, lumps/bumps, pain/tenderness, redness, and swelling, categorized according to severity grades (mild, moderate, severe).

2. Supplementary Tables

Table S1. Investigator GAIS results.

	Month 1	Month 4
	N (%)	N (%)
N (MV)	86	85
Responder	77 (90%)	69 (81%)
Not responder	9 (10%)	16 (19%)

Table S2. Subject GAIS results.

	Month 1	Month 4
	N (%)	N (%)
N (MV)	86	85
Responder	69 (80%)	62 (73%)
Not responder	17 (20%)	23 (27%)

Table S3. Skin texture parameters parameter (i.e., Uf and Ur) values for each subject.

Subject #	Uf D0	Ur D0	Uf M1	Ur M1	Uf M4	Ur M4
G1-001	0.301	0.087	0.276	0.098	0.262	0.122
G1-002	0.486	0.128	0.241	0.073	0.346	0.114
G1-003	0.4	0.144	0.323	0.124	0.332	0.147
G1-004	0.333	0.143	0.331	0.142	0.353	0.178
G1-005	0.388	0.118	0.352	0.09	0.387	0.128
G1-006	0.288	0.097	0.246	0.093	0.298	0.099
G1-007	0.513	0.149	0.471	0.174	0.441	0.149
G1-008	0.341	0.12	0.314	0.106	0.333	0.12
G1-009	0.34	0.066	0.305	0.087	0.366	0.126
G1-010	0.556	0.115	0.396	0.134	0.41	0.165
G1-011	0.384	0.14	0.312	0.131	0.354	0.196
G1-012	0.384	0.132	0.358	0.092	0.392	0.114
G1-013	0.481	0.104	0.407	0.1	0.381	0.086
G1-014	0.305	0.078	0.306	0.082	0.29	0.105
G1-015	0.544	0.303	0.445	0.217	0.428	0.215
G1-016	0.255	0.095	0.261	0.1	0.246	0.111
G1-017	0.45	0.142	0.365	0.141	0.393	0.166
G1-018	0.327	0.155	0.402	0.231	0.403	0.206

Subject #	Uf D0	Ur D0	Uf M1	Ur M1	Uf M4	Ur M4
G1-019	0.316	0.129	0.315	0.158	0.306	0.176
G1-020	0.282	0.091	0.302	0.084	0.254	0.094
G1-021	0.303	0.112	0.268	0.111	0.247	0.103
G1-022	0.3	0.109	0.283	0.116	0.348	0.132
G1-023	0.32	0.135	0.352	0.147	0.297	0.133
G1-024	0.369	0.165	0.388	0.151	0.389	0.171
G1-025	0.482	0.197	0.476	0.218	0.501	0.289
G1-026	0.374	0.111	0.407	0.124	0.355	0.121
G1-027	0.434	0.226	0.43	0.18	0.419	0.222
G1-028	0.367	0.108	0.369	0.101	0.39	0.097
G1-029	0.369	0.132	0.407	0.163	0.335	0.147
G1-030	0.389	0.096	0.52	0.144	0.446	0.12
G1-031	0.351	0.106	0.343	0.097	0.369	0.126
G1-032	0.243	0.102	0.278	0.1	0.262	0.118
G1-033	0.357	0.154	0.343	0.128	0.368	0.153
G1-034	0.285	0.147	0.284	0.092	0.272	0.106
G1-035	0.296	0.109	0.292	0.102	0.311	0.126
G1-036	0.299	0.107	0.305	0.109	0.291	0.116
G1-037	0.332	0.135	0.406	0.132	0.318	0.142
G1-038	0.376	0.094	0.377	0.111	0.391	0.118
G1-039	0.379	0.157	0.409	0.176	0.367	0.162
G1-040	0.329	0.081	0.347	0.093	0.37	0.106
G1-041	0.353	0.125	0.387	0.132	0.364	0.128
G1-042	0.297	0.097	0.297	0.088	0.326	0.123
G1-043	0.301	0.14	0.324	0.14	0.322	0.143
G1-044	0.422	0.152	0.417	0.127	0.405	0.152
G1-045	0.342	0.117	0.34	0.097	0.393	0.122
G1-046	0.453	0.263	0.479	0.272	0.56	0.307
G1-047	0.259	0.095	0.296	0.077	0.268	0.111
G1-048	0.34	0.134	0.31	0.112	0.352	0.127
G1-049	0.285	0.115	0.354	0.125	0.292	0.121
G1-050	0.463	0.207	0.453	0.19	0.425	0.185
G1-051	0.253	0.098	0.284	0.105	0.301	0.119
G1-052	0.265	0.081	0.288	0.07	0.302	0.095

Subject #	Uf D0	Ur D0	Uf M1	Ur M1	Uf M4	Ur M4
G1-053	0.327	0.151	0.315	0.103	0.35	0.13
G1-054	0.279	0.122	0.301	0.093	0.31	0.122
G1-055	0.343	0.12	0.305	0.123	0.345	0.122
G1-056	0.602	0.254	0.601	0.271	0.48	0.171
G1-057	0.447	0.265	0.385	0.192	0.371	0.169
G1-058	0.294	0.097	0.294	0.087	0.317	0.124
G1-059	0.346	0.118	0.421	0.157	0.335	0.098
G1-060	0.342	0.115	0.31	0.082	0.368	0.091
G1-061	0.347	0.156	0.403	0.124	0.336	0.13
G1-062	0.395	0.135	0.385	0.147	0.404	0.139
G1-063	0.348	0.173	0.368	0.164	0.351	0.162
G1-064	0.305	0.126	.	.	.	.
